# Exploring In Vivo Dynamics of Bovine Milk Derived Gangliosides

**DOI:** 10.3390/nu12030711

**Published:** 2020-03-07

**Authors:** Welma Stonehouse, Bradley Klingner, Paul McJarrow, Bertram Fong, Nathan O’Callaghan

**Affiliations:** 1 Commonwealth Scientific Industrial Research Organisation, Adelaide 5000, Australia; Bradley.Klingner@csiro.au (B.K.);; 2 Fonterra Co-Operative Group, Palmerston North 4414, New Zealand; Paul.McJarrow@fonterra.com (P.M.); Bertram.Fong@fonterra.com (B.F.)

**Keywords:** gangliosides, diurnal variation, day-to-day variation, postprandial, acute, serum, plasma, women of child bearing age, storage time, storage temperature

## Abstract

Gangliosides are glycosphingolipids present in mammalian cell membranes, playing important structural and functional roles. Human studies on the health benefits of gangliosides are increasing, but knowledge gaps regarding ganglioside analysis exist. The study aimed to investigate blood sample type (serum/plasma), storage conditions, diurnal, day-to-day variation and acute effects of consuming bovine-derived gangliosides on circulating monosialylated gangliosides. Seventy-one women (18–40 yrs, 20–≤30.0 kg/m^2^) were enrolled and 61 completed the intervention. They visited the clinic three times following overnight fasting. Serum/plasma gangliosides were analyzed over 2 h (visit-1), 8 h (visit-2) and 8 h following either zero or high ganglioside meals (visit-3). Samples stored at −20 °C and −70 °C were analyzed at 3-, 6-, 12- and 18-months. Plasma and serum GM3-gangliosides did not differ, plasma GM3 did not change diurnally, from day-to-day, in response to a high vs. low ganglioside meal or after 7-days low ganglioside vs. habitual diet (*P* > 0.05). GM3 concentrations were lower in samples stored at −70 °C vs. −20 °C from 6-months onwards and decreased over time with lowest levels at 12- and 18-months stored at −70 °C. In conclusion, either serum/plasma stored at −20- or −70 °C for up to 6 months, are acceptable for GM3-ganglioside analysis. Blood samples can be collected at any time of the day and participants do not have to be in the fasted state.

## 1. Introduction

Gangliosides are complex sialic acid containing glycosphingolipids linked to a hydrophilic sugar chain and hydrophobic ceramide [1]. The nomenclature devised by Svennerholm [2], rely on the degree of sialylation and glycosidic linkages giving rise to different classes of gangliosides based on two letters and one subscript number (e.g., GM3, GD3 or GT3). All gangliosides start with “G”; the second letter indicates the number of sialic acid monosaccharides (e.g., “M”, “D”, “T” or “Q” refers to mono-, di-, tri- or quad-residues); and the number corresponds to five minus the number of sugar moieties present in the molecule. In some cases, a lowercase letter at the end indicates where the sialic acid residues are attached. Gangliosides are further differentiated by a variety of fatty acids linked to the ceramide (e.g., GM3 34:1) [2]. 

Gangliosides occur naturally in human diets, other than vegan, and are high in foods from animal origins such as dairy products, meats and eggs, and particularly high in human breast milk [3,4]. They derive their name from brain ganglion cells from which they were initially isolated. Today we know that gangliosides are present in all mammalian cell membranes and are particularly abundant in neural cell membranes, comprising 10% of the total lipid mass in brain [5]. From their positioning in membrane microdomains, including lipid rafts and caveolae, they play important structural and functional roles and have been implicated in regulating cell–cell interactions and modulating signal transduction [5]. Ganglioside profiles differ significantly between tissues and stages of development [6]. The role of each individual ganglioside is not yet clarified but it is generally assumed that gangliosides play important roles within the tissues they appear. Gangliosides are consumed in the form of GM3 and GD3 which reaches the intestinal tract mostly intact [4]. Once absorbed in the intestine, they may be remodeled in enterocytes inducing changes in total membrane ganglioside content [6]. GM3 and GD3 are the dominant gangliosides in gut tissue while GM3 is the dominant ganglioside in the circulation of humans [6] and favored for placental transfer [4]. The major gangliosides in the brain are GM1, GD1a, DG1b and GT1b (representing >90% of gangliosides) [7]. A growing body of scientific evidence is emerging linking dietary gangliosides to a variety of bioactivities such as neurological development, intestinal maturation, intestinal immunity development and gut-barrier function [1,4,8,9,10].

However, despite an increase in research activity demonstrating the health benefits of dietary gangliosides, key knowledge gaps, important for designing robust human clinical studies, exist that limit their application and utilization. These methodological gaps include best approaches regarding blood sample type and storage of blood samples for ganglioside analysis, the normal diurnal and day-to-day variation in circulating gangliosides as well as the acute impact of consuming bovine milk derived gangliosides on circulating levels of gangliosides in humans. Addressing these knowledge gaps will inform the time of day blood samples for ganglioside analysis should be taken; whether participants must be fasted; optimal sample type and time frame from sample taking to analysis to ensure valid results. A large body of scientific evidence, although mostly in pre-clinical models, supports ganglioside’s role in neurological development and function [7]. Hence, life stages during pregnancy and lactation to investigate fetus and infant outcomes is an important target population for ganglioside clinical trials. The study aimed to address these key knowledge gaps. Specific objectives included: (1) to compare serum vs. plasma for quantifying blood ganglioside levels; (2) to determine the stability of frozen serum/plasma gangliosides stored at different temperatures (−20 °C and −70 °C) and over time post-sampling (at 3, 6, 12- and 18-months); (3) to measure how ganglioside concentrations vary from fasting state over the course of 8 h; (4) to measure how ganglioside concentrations vary from day to day; and (5) to determine whether recent intake (habitual vs. low ganglioside diet over 7 days) and acute intake of a high compared to a low ganglioside meal will measurably and significantly increase circulating concentrations of gangliosides postprandially in healthy women of child bearing age. GM3 gangliosides were investigated as they are the dominant ganglioside in the circulation [6].

The following hypotheses were tested: (1) GM3 ganglioside levels in blood samples stored as either plasma or serum will not differ; (2) longer storage duration and increased storage temperature conditions will result in lower GM3 ganglioside concentrations in serum/plasma; (3) circulating GM3 ganglioside concentrations change throughout the day due to diurnal variation in endogenous synthesis; (4) circulating GM3 ganglioside concentrations do not vary from day to day; and (5) and bovine gangliosides are absorbed and enter the circulation after consumption in women of child bearing age resulting in increased circulating levels.

## 2. Materials and Methods 

The study was conducted at CSIRO’s Nutrition and Health Research Clinic, Adelaide, South Australia in two phases. Phase 1 investigated the optimal sample type and storage conditions. Phase 2 included a diurnal variation study involving the monitoring of circulating gangliosides over the course of a day and from day-to-day and a randomized controlled acute trial investigating circulating gangliosides after consuming a high- vs. low-ganglioside meal. 

Phases 1 and 2 were approved by the CSIRO Health and Medical Human Research Ethics Committee (Phase 1: Low Risk Review Panel, reference no. 8/2017; Phase 2: reference 03/2017). Furthermore, the phase 2 study was registered at anzctr.org.au as ACTRN 12617001006336. Oral and written information about the study objectives and protocols were given to each eligible participant and participants provided written, informed consent to the study protocol prior to their participation. Phase 1 was conducted between May 2017 and November 2018 and phase 2 between July and October 2017. 

### 2.1. Blood Sampling and Storage for Ganglioside Analysis

One-off blood samples were collected from 10 healthy female participants (in- and exclusion criteria were the same as described below) at the CSIRO Nutrition and Health Research Clinic. Blood samples were collected following an overnight fast and one hour after consuming a high ganglioside meal (See Section 2.2.3 for details regarding the high ganglioside meal). 

### 2.2. Circulating Ganglioside Dynamics

#### 2.2.1. Study Population

Seventy-one healthy female participants aged 18–40 years, body mass index (BMI) 20–≤30.0 kg/m^2^ were enrolled for the study. The target population was chosen to represent a study population most likely to be targeted for ganglioside studies, namely pregnant women and mothers to investigate fetus or infant outcomes. As there is little known about the in vivo uptake kinetics of gangliosides from the diet, the experiments were conducted on non-pregnant/lactating women of child-bearing age rather than pregnant/lactating women. Participants were recruited from the CSIRO Nutrition and Health Research clinic database, through the CSIRO Nutrition and Health Research clinic website, Facebook advertisements and advertising within local academic institutions. Potential participants were screened and excluded for the following conditions: known history or presence of chronic disease—cancer, type 2 diabetes, heart disease, gastrointestinal disease, renal or hepatic disease—pancreatic insufficiency, stomach ulcers, drug abuse or alcoholism, thyroid disorders or any condition that may, in the opinion of the principle investigator, influence the study outcomes; known food allergies, hypersensitivity, dietary avoidance or intolerance to the study foods; taking medications known to influence lipid metabolism, glucose tolerance and gastric emptying (oral contraceptives were accepted); sufferers of bleeding disorders; pregnancy or breastfeeding (self-reported); persons considered by the investigator to be unwilling, unlikely or unable to comprehend or comply with the study protocol and participation in another research study within 30 days preceding the start of this study. 

#### 2.2.2. Study Design and Intervention

**Figure 1:**
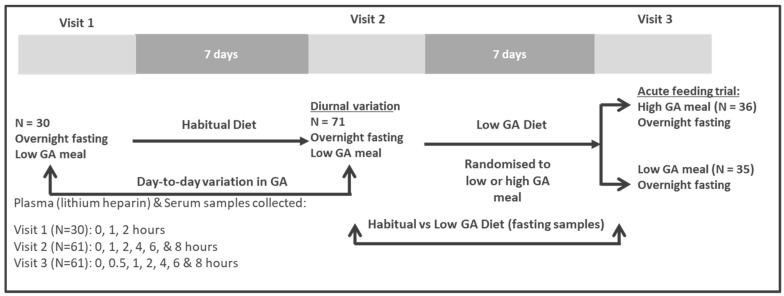
Study design. GA, gangliosides.

The study design is illustrated in Figure 1. Visit 1: Participants (*n* = 30, randomly selected from total sample of 71) presented to the CSIRO Nutrition and Health clinic between 7 and 9 am, following an overnight fast, and remained within the clinic for the next 2 h. Participants had an IV Cannula inserted for the duration of this visit. A fasting baseline blood sample (6 mL) was collected, after which participants were provided with a low ganglioside meal. Further blood samples (6 mL) were subsequently collected at 1 h and 2 h. Day-to-day variability in plasma gangliosides were determined by comparing these samples to the fasting, 1 and 2 h samples collected at visit 2.

Visit 2: Participants (*n* = 71) followed their habitual diet for 7 days and presented to the CSIRO Nutrition and Health clinic between 7 and 9 am following an overnight fast. They remained within the clinic for the next 8 h. Participants had an IV Cannula inserted for the duration of this visit. A fasting baseline blood sample (6 mL) was collected after which participants were provided with low ganglioside meals and snacks throughout the day (breakfast, lunch as well as mid-morning and mid-afternoon snacks). Subsequent plasma samples (6 mL) were collected at 1 h, 2 h, 4 h, 6 h and 8 h. Fasting samples from this visit were compared to fasting samples from visit 3 to assess how plasma ganglioside concentrations change after following a low ganglioside diet vs. habitual diet for 1 week.

Visit 3: The acute ganglioside intervention was conducted using a randomized parallel study design. During the week preceding the test day participants were requested to consume a low ganglioside diet designed by a dietitian. On the clinic test day participants presented to the CSIRO Nutrition and Health Research Clinic between 7 and 9 am following an overnight fast. The participants then remained at the clinic for the next 8 h. Participants were randomly assigned by computer sequence generation (http://www.randomisation.com) to interventions including consumption of a high ganglioside meal or a low ganglioside meal. The random allocation sequence was generated by a staff member not involved with entering participants into the trial to ensure allocation concealment. For the duration of the clinic test day, an IV cannula was inserted into the participants arm. A fasting blood sample (6 mL) was collected, after which participants consumed their test meal. Participants were allowed up to 15 min to consume the test meal. Subsequent blood samples (6 mL) were collected at 0.5 h, 1 h, 2 h, 4 h, 6 h, 8 h. Throughout the day participants were permitted to consume and were provided with low ganglioside, low fat snacks and meals. Apart from the study specific dietary changes, participants were requested to maintain their habitual lifestyle patterns throughout the duration of the study. 

Treatments were fully concealed from staff responsible for allocation. Staff responsible for sample and statistical analysis were also blinded to the treatments until after statistical analysis. However, the participants were not blinded to treatments as it was possible to identify the milk type they were consuming. 

#### 2.2.3. Assessment of Dietary Intake, Dietary Intervention and Low Ganglioside Diet

Participant’s usual (habitual) dietary intake was assessed prior to commencing the low ganglioside diet using the Automated Self-Administered 24-h (ASA24-Australia) Dietary Assessment Tool, version (2016), developed and validated by the National Cancer Institute, Bethesda, MD [11]. Two 24-h recalls were collected, the first at visit 1 under supervision by a dietitian and the second at home within 7 days of the first visit randomly covering all days of the week including weekends. Nutrient analysis is automated through the ASA24 program and uses the Australian Food, Supplement and Nutrient Database (AUSNUT, 2011-13).

The high ganglioside intervention meal consisted of 23 g ganglioside-enriched milk powder (SureStart^TM^ MFGM Lipid 100 [485 mg gangliosides/100 g powder], supplied by Fonterra Co-operative Group, Palmerston North, New Zealand), reconstituted with 200 mL of water to deliver 112 mg gangliosides per meal (98% GD3 and 2% GM3 [analyzed by Fonterra Co-operative Group]). To put this in context, daily intake of gangliosides by a healthy Canadian population who consumed egg, tuna, beef, milk and milk products (yoghurt and cheese) was estimated at <200 mg/day per 2000 kcal diet [12,13]. The low ganglioside intervention meal consisted of 200 mL of soy milk (VitaSoy Soy Milky), devoid of gangliosides and the most nutritionally comparable to bovine milk. 

During clinic visits, low ganglioside meals and snacks were provided to participants to consume ad libitum throughout the day. Choices included soy or almond milk, breakfast cereals, bread, margarine, spreads (vegemite, peanut butter, jam and honey), muesli bars, fresh fruit, popcorn, a range of hot meals which were specially prepared for the study using only low-ganglioside ingredients (e.g., vegan fried rice, tomato-based pasta, vegetable soup). 

Participants were requested to follow a low ganglioside diet 7-days before the acute feeding trial. To enhance compliance, participants were counselled on this eating pattern by dietitians during visit 1. The dietitian discussed with each participant their usual dietary intake and substitutions which could be made to modify this to be low ganglioside. Specifically, participants were instructed to avoid consuming any meat (red meat, chicken, fish and other seafood) or dairy products (milk, yoghurt, cheese, cream, custard, butter). In addition, participants were provided with vouchers to the value of $70 and a shopping list/recipe booklet to further enhance compliance. Participants were provided with a checklist to record any non-compliance to the low ganglioside diet and were asked to report on accidental consumption of any animal foods (date of occurrence, type, amount) and return the checklist at their subsequent visit.

#### 2.2.4. Blood Sampling and Ganglioside Analysis

Venous blood samples were collected into vacutainers containing lithium heparin for the preparation of plasma. Blood for serum samples were collected in tubes containing clot activator and allowed to clot at room temperature for 30 min before processing. Plasma and serum were prepared by centrifugation (GS-6R centrifuge; Beckman Coulter Inc., Brea, CA, USA) at 2095 g for 10 min at 4 °C, within 30 min of collection (for plasma samples). For phase 1, serum and plasma samples were stored at −20 and −70 °C in the clinic laboratory until analysis at 3, 6, 12 and 18 months (only plasma analyzed at 18 months) post-sampling. For phase 2, samples were stored at −70 °C for less than 4 months prior to analysis. 

Electrospray liquid chromatography tandem mass spectrometry (ESI-LC MS/MS) was performed using an API 4000 QTrap mass spectrometer (Sciex, Concord, Canada). Liquid chromatographic separation was by means of an ACQUITY LC system (Waters, Milford, MA, USA) equipped with a 2.1 mm ID x 50 mm BEH C18 reversed phase analytical column with a 1.7 µm particle size (P/N: 186002350,S/N: 02503563315745, Lot: 0250350331). 

LC conditions: A binary solvent system was used with mobile phase A consisting of 10% methanol/90% water in 1 mM ammonium acetate and mobile phase B consisting of 100% methanol in 1 mM ammonium acetate. 10 µL of sample was loaded on the column at a flow rate of 300 µL/min using 90% B. Chromatographic separation was performed using a gradient from 90% B to 95% B from 0.07 min to 2.73 min, held at 95% B until 4.07 min, increased to 99.9% B linearly to 4.13 min and held until 6.13 min at a flow rate of 300 µL/min. The column was then re-equilibrated from 6.13 to 7.00 min with 90% B. Flow was diverted to waste for 0.5 min after the sample injection. Column temperature was 50 °C and total time for each sample to run was 7 min.

MS conditions: Data were acquired in multiple reaction monitoring (MRM) mode. Quantification using peak areas for the gangliosides and the corresponding d_3_ internal standard were calculated using Analyst 1.6.2 software (Sciex, Concord, Canada). Retention times and MRMs are summarised in Supplementary Table S1 and a chromatogram illustrating MS-data can be seen in Supplementary Figure S1.

In order to reduce analytical variability due to inter-run variability in phase 2, all samples from an individual were analyzed in the same analytical run.

Most GD, GM1 and GM2 ganglioside species were below the limit of detection using the methodology and equipment as described. Serum and plasma GM3 ganglioside concentrations are reported. 

### 2.3. Statistical Analyses

Relevant data to estimate an effect size and SD (of difference) within the target population was not available for a sample size calculation. In the absence of this information the sample size was based on detecting statistical significant differences in GM3 (the dominant circulatory ganglioside [14]) between the high and low ganglioside meals. Using G*Power 3.1.9.2 it was calculated that a sample size of 30 participants per group (60 in total) will provide 80% power (α 0.05, two-tailed, SD = 5.4 mg/L based on data from maternal serum concentrations [15]) to detect a significant difference between groups of 4 mg/L in serum ganglioside concentrations. Ma et al. [15] detected a difference of >5 mg/L between serum collected at the second and third trimesters of pregnancy, hence this difference is physiologically possible. A total sample of 71 participants were enrolled to account for attrition during the study.

Plasma and serum samples were compared using Paired Samples *T*-Test. Differences between storage times and temperatures were analyzed using linear mixed model analysis of variance. An unstructured repeated covariance matrix structure was used. Time and temperature were included as fixed factors and analyzed for main effects and time*temperature interaction effects. Post-hoc analysis was performed adjusting for multiple comparisons using Bonferroni adjustments. Some variables were log transformed to achieve greater model validity. Outcome variables were examined for normality using Kolmogorov–Smirnov, Shapiro–Wilk tests and normality plots. Non-normally distributed data were transformed into approximate normal distributions by logarithmic transformation when required. 

Day-to-day differences, diurnal variation and differences between the acute effects of low- and high ganglioside meals on plasma GM3 ganglioside concentrations were analyzed using mixed effects linear models. Each participant was treated as fixed effects in the repeated measures model, allowing each participant to have their own intercept and slopes that enables more precise modelling of longitudinal changes. An unstructured repeated covariance matrix structure was used. For analysis of day-to-day differences, time (0, 1 and 2 h) and day (visit) (day 1 and day 2) were included as fixed factors and analyzed for main effects and time x visit interaction effects. For analysis of diurnal variation (hourly differences over 8 h), time was included as fixed factor and analyzed for main effect of time. For acute differences over 8 h between low and high ganglioside meals, time (0, 0.5, 1, 2, 4, 6, 8 h) and meal (low vs. high ganglioside meal) were included as fixed factors and analyzed for main effects and time x meal interaction effects. In cases where significant (*P* < 0.05) main or interaction effects were seen post-hoc analysis were performed with Bonferroni adjustments in order to determine which time points differed significantly. 

Differences in fasting plasma GM concentrations after consuming a habitual diet vs. a low ganglioside diet were determined using Paired Student *T*-Test. 

Statistical analysis was performed using SPSS Software version 25 (IBM Corporation, New York, USA). Results are presented as means and SD of the raw data. For all analyses, statistical significance was determined at a *P*-value of < 0.05. 

## 3. Results

### 3.1. Ganglioside Analysis Repeatability

The intra-run coefficient of variance (CV) for GM3 species ranged from 9% to 18%; inter-run CV ranged from 14% to 26%. Data reported is for GM3 gangliosides, the main ganglioside in the circulation [6].

### 3.2. Blood Sampling and Storage for Ganglioside Analysis

GM3 ganglioside concentrations did not differ between serum and plasma samples taken either after an overnight fast or 60 min after a high ganglioside meal (Table 1) or stored at different temperatures or times (data not shown). The statistically significant differences seen for GM3 38:2 at T0 and GM3 34:2 at T60 were likely due to chance as a result of multiple statistical tests performed. Individual results also did not show any specific patterns (Supplementary Figure S2).

**Figure 2:**
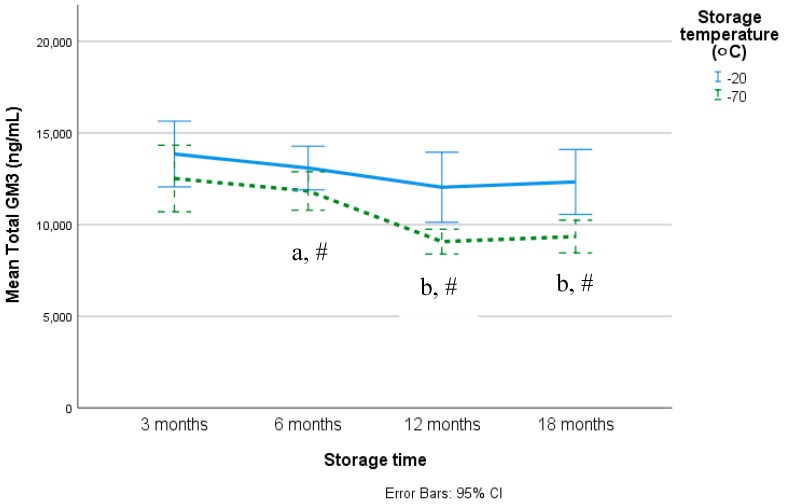
Mean (95% CI) changes in plasma total GM3 gangliosides stored over time at −20 °C and −70 °C. Different symbols differed significantly, ^a,b^—post-hoc analysis over storage times; ^*,#^—post-hoc analyses over storage temperatures.

Mean (SD) differences in plasma GM3 gangliosides between different storage times and storage temperatures are summarized in Figure 2 and Table 2. As no differences were seen between serum and plasma GM3 concentrations, analyses were conducted on plasma. As no differences were seen between T0 and T60, data presented are for T0 only. Storage at −70 °C resulted in significantly lower plasma GM concentrations for most species compared to storage at −20 °C. The lower concentrations became significant from 6 months and onwards for total GM3 and several other GM3 species (40:2, 40:1, 42:2, 42:1, time*temperature interactions *P* < 0.05). 

In terms of storage over 18 months, plasma GM3 concentrations decreased over time for total GM3 and most GM3 species (34:1, 36:2, 36:1, 38:2, 38:1, 40:1). For most, the decrease was significant from 12 months with no differences seen between 12 and 18 months, while for total GM3 the decrease was only significant for those samples stored at −70 °C (time*temperature interactions *P* < 0.05). This is also evident when looking at the individual’s plasma total GM3 changes (Supplementary Figure S3). Plasma GM3 C42:2 showed inconsistent patterns of change over time, whereas GM3 32:1 and GM3 40:2 increased over time. 

Analysis of plasma taken at T60 and serum at T0 and T60 up to 12 months, showed the same inconsistent, non-significant patterns of change (Data not shown). 

### 3.3. Circulating Ganglioside Dynamics

**Figure 3:**
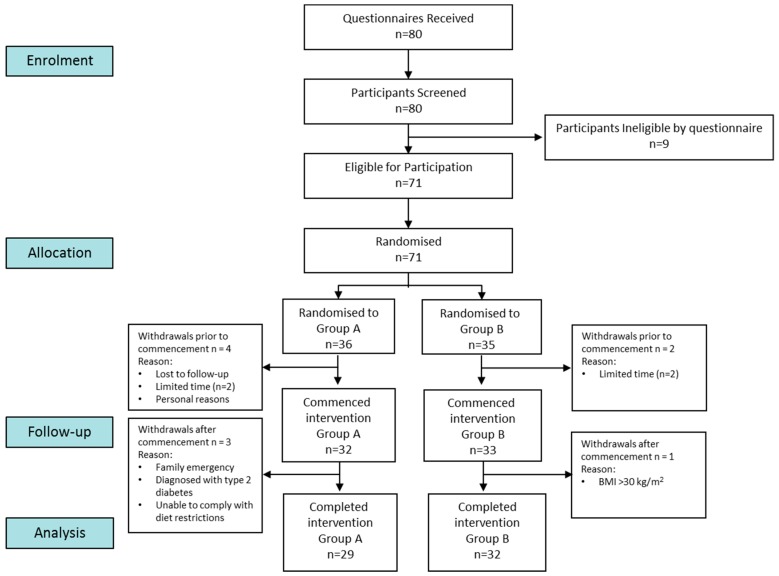
Flow diagram of participants through the trial (Group A = high ganglioside meal; Group B = low ganglioside meal).

Eighty volunteers attended a clinic screening visit and 71 eligible participants were enrolled in the study (Figure 3); 61 participants completed the intervention.

The high ganglioside meal was safe and well tolerated by participants as no adverse events related to the intervention products were reported. 

Diurnal variation results are summarized in Table 3. Total mean plasma GM3 did not change over the course of 8 h. Large variations were seen between individuals over time, but individual’s results tended to stay in the top, middle or bottom thirds of the distribution (Supplementary Figure S4). Plasma GM3 40:1 and GM3 42:2 increased slightly after 1 h compared to baseline (t0) and mean plasma GM3 42:1 was significantly higher after 6 h compared to baseline (t0).

The day-to-day variability in plasma GM3 concentrations are summarized in Table 4. Plasma concentrations did not differ between day 1 and day 2 for baseline and 1-h samples, but the 2-h point differed significantly between days for total GM3, GM3 32:1, GM3 34:1, GM3 36:2 and GM3 38:1. The differences in days at 2 h were driven by unexplained significant increases in concentrations on day 1 between baseline and 2 h, whereas this increase was not observed on day 2 (Table 4). Considering that the day-to-day differences were only seen at one occasion, plasma GM3 gangliosides likely do not vary from day-to-day. 

Total fasting plasma GM3 concentrations did not differ between consuming a habitual diet vs. a low ganglioside diet for a week (Table 5). Small inconsistent differences were seen for some individual GM3 species. Plasma GM3 32:1, GM3 36:2 concentrations were lower after the low ganglioside diet compared to the habitual diet whereas plasma GM3 40:2, GM3 40:1, GM3 42:2 and GM3 42:1 concentrations were higher after the low ganglioside diet vs. habitual diet (Table 5).

Results from the acute feeding trial are summarized in Table 6. Consumption of a high ganglioside meal did not acutely affect plasma total GM3 compared to a low ganglioside meal over 8 h (Table 6). Large variations were seen between individuals and no clear distinction is apparent between individuals following the high compared to the low ganglioside meals (Supplementary Figure S5). The main effects of time were seen for some plasma GM3 species. Lower plasma concentrations were seen over time compared to fasting concentrations (T0) for GM3 34:1, GM3 36:1, GM3 38:2 and GM3 40:2. A similar trend was seen for plasma GM3 36:2 concentrations with concentrations after 4 h significantly lower compared to 1 h. 

## 4. Discussion

The study investigated, for the first time, comparisons between serum and plasma for quantifying blood ganglioside levels, stability of frozen plasma gangliosides stored at different temperatures (−20 °C and −70 °C) and over time post-sampling (at 3, 6, 12- and 18-months), the normal diurnal and day-to-day variability in plasma gangliosides as well as the acute impact of consuming a meal high in bovine derived gangliosides on circulatory levels of gangliosides. Results for GM3 gangliosides, the major ganglioside in the circulation, were reported. 

The main findings from the series of experiments were that GM3 ganglioside concentrations did not differ between serum and plasma samples. Concentrations were lower in samples stored at −70 °C compared to −20 °C which became statistically significant from 6 months and onwards for total GM3 and most species. GM3 ganglioside concentrations decreased over time with lowest levels at 12 and 18 months for most species. For total GM3, the decrease over time was only significant for samples stored at −70°C. Plasma GM3 ganglioside concentrations were mostly stable over the course of an 8-h day and from day-to-day. Recent changes in diet (low ganglioside diet over 7 days vs. habitual diet) did not affect fasting total plasma GM3 ganglioside concentrations and neither did acute intake of a high ganglioside meal vs. a low ganglioside meal affect GM3 ganglioside concentrations over 8 h. Although significant differences occurred in some individual GM3 ganglioside species over the course of a day, these may have occurred by chance considering multiple statistical analyses (Type 1 error). To support this; time main effects during the diurnal experiment were inconsistent with the time main effects during the acute trial, with different GM species changing over the two experiments and in opposite directions. For example, plasma GM3 40:1, GM3 42:2, GM3 42:1 increased during diurnal variation, but not during the acute trial; whereas GM3 34:1, GM3 36:1, GM3 38:2, GM3 40:2, GM3 36:2 decreased during the acute trial, but not during the diurnal trial. 

The results convincingly demonstrate that either serum or plasma (specifically lithium heparin plasma) can be used for GM3 ganglioside analysis. This was consistently shown under various conditions (samples taken after fasting or 60 min after a high ganglioside meal or stored at −20 °C or −70 °C). Plasma is recommended as the preferred matrix as it gave a better mass spec response under experimental conditions used in the current experiment. Lithium heparin plasma has also previously been shown to be the best choice of anticoagulant for plasma metabolomics [16].

The lower GM3 ganglioside concentrations in plasma at −70 °C vs. −20 °C was unexpected. Other lipids, such as fatty acids, cholesterol, triglycerides and apolipoproteins in serum/plasma stored at temperatures ≤60 °C are generally stable [17,18,19]. In fact, Metherel and Stark, in their review of the literature, concluded that plasma/serum polyunsaturated fatty acids (PUFA) remained stable for up to 10 years at sub-zero temperatures [19]. Gangliosides, on the other hand, are less stable due to the lability of the sialic linkage as a 2,3 or 2,6 compared to triglycerides [20]. However, it remains unclear why plasma concentrations were lower when stored at −70 °C than when stored at −20 °C. Other non-sample storage related factors that may have affected storage at −70 °C have been ruled out. For example, the −70 °C freezer at the CSIRO Nutrition and Health Research Clinic is continuously monitored and recorded and no temperature excursions occurred over the storage period. It was considered whether day-to-day laboratory variation could have contributed to the findings. The lack of an external standard limits judgement in this regard. However, since the same patterns of changes over time and between temperatures were seen for plasma taken at T60 and serum at T0 and T60 (up to 12 months), it is unlikely to be the case. Some GM3 species (GM3 32:1, GM3 40:2) showed increases over time. This anomaly is likely due to analytical variation as these are minor species in the circulation and low concentrations generally leads to greater variability and greater chance of false positives. Indeed, inter-run CV’s for these species ranged from 19%–23%. Before any conclusions can be drawn regarding the stability of plasma gangliosides at −70 °C vs. −20 °C, it will be important to investigate whether the lower concentrations at −70 °C may have been due to factors related to analytical method. It is speculated that the protein precipitation extraction method used in the current study, under lower freezing conditions, may have resulted in removal of gangliosides with the denaturation of proteins. Furthermore, sample defrosting time and temperature may have affected the results. Due to the complex structure of gangliosides, consisting of hydrophobic and hydrophilic domains that may interact, storage at −70 °C may affect their ability to dissolve at the defrosting temperature and times used in the current experiment (thawed to room temperature on the bench). Lower GM ganglioside concentrations at −70 °C vs. −20 °C were seen at all time points, although the difference was not significant at 3 months. Further exploration of potential reasons for lower ganglioside concentrations in samples stored at −70 °C is needed. 

A limitation of the current work, however, is that analysis was not conducted at zero time points. Before final recommendations can be made, it is recommended that future work include a zero-time point (analysis conducted within a week of blood collection). It is recommended that the experiment is repeated once any potential effects due to analytical method has been ruled out. For prospective intervention or observational studies longer than 6 months, it seems to be better to analyze samples after collection or within 6 months and not to store samples until the end of the trial and analyze in one batch, as is often done to reduce day-to-day laboratory variability. 

The optimal range for serum/plasma GM3 in humans is unknown due to a limited number of human studies reporting links between serum/plasma GM3 and health outcomes, a lack of standardization of ganglioside methodology across laboratories and methodological gaps like those investigated in the current study. A limited number of studies to date have measured gangliosides in human serum/plasma and in most cases the analysis was done on one-off samples and in some studies after chronic intake of gangliosides. Levels reported varied considerably (values were converted to mg/L for the purpose of comparison); in the current study in women of child-bearing age mean fasting plasma GM3 was ~6.65 (SD 1.47) mg/L; in normal adults, plasma concentrations of 9.72 (SD 3.68) mg/L [21] and ~0.78 (SE 0.1) mg/L [10] were reported; in infants serum GM3 concentrations ranged from 7.27 (SD 2.15) mg/L (formula fed) to 9.91 (SD 2.48) mg/L (breastmilk fed) [22]; and in pregnant women serum concentrations ranged from 13.1 (SD 5.4) mg/L (2^nd^ trimester) to 18.4 (SD 9.2) mg/L (3^rd^ trimester) [15]. These differences were most likely due to differences in analytical methods. In addition, plasma GM3 34:1 was the most abundant GM species in plasma (39% of total GM3), like Miklavcic et al. [10]. 

The literature has several examples of chronic intake of enriched ganglioside diets influencing blood ganglioside levels in humans and animals. Gurnida et al. (2012) reported, after 16–22 weeks of supplementation of infants with ganglioside enriched infant formula, serum GM3, GD3 and total gangliosides increased significantly compared to standard infant formula (plasma GM3: 9.04 (95%CI 8.22, 9.85) vs. 7.27 (95%CI 6.46, 8.02) µg/mL) [22]. Miklavcic et al. [10] showed a significant increase in plasma GD3 in healthy volunteers consuming 43 g/day of gangliosides (containing 80% GD3, 20% GM3) for 8 weeks, but no changes were seen in plasma GM3 concentrations. Park et al. [6] reported significant increases in total plasma ganglioside in rats fed a ganglioside enriched diet comprising 80% GD3 for 2 weeks compared to a control diet, but no changes were seen in individual gangliosides including GM3 (plasma GD3 was undetected). In contrast to human serum/plasma, where GM3 is the major ganglioside, they showed that GD1a was the major ganglioside in rat plasma.

Studies assessing circulatory gangliosides in the postprandial state in humans are sparse, limiting comparison with existing evidence. The only other study that the authors are aware of is the study by Meikle et al. [23] who showed a significant increase in plasma GM3 gangliosides after consumption of a high dairy meal (consisting of 60 g cheddar cheese, 20 g butter, 300 mL extra creamy whole milk with toast) vs. a soy-based meal. It is important to note that Meikle et al. used a non-specific lipidomic approach in a small sample (*n* = 16), which is less robust than the current study. 

There may be several explanations why plasma GM3 gangliosides were not affected by the high ganglioside meal in this study. Although GM3 was present in minor amounts (2%) while the major ganglioside in the meal was GD3 (98%), previous studies have shown that chronic feeding with high GD3 meals resulted in increased GM3 levels. Gurnida et al., used a ganglioside intervention with a similar composition than the current study and showed increases in total ganglioside levels with the largest increase in GM3 in infants after chronic supplementation [22]. GM3 is the dominant ganglioside in the circulation [6] and seems to be strictly regulated [10]. The presence of glycosphingolipid-metabolizing enzymes in the brush-border membrane suggest that dietary gangliosides may undergo conversion to other ganglioside species in the gut before absorption [10]. Furthermore, gangliosides may undergo remodeling in enterocytes and released in the circulation as newly formed ganglioside [4]. GM3 may also be broken down to lactosylceramide [4,6]. It is also possible that the metabolism of dietary gangliosides in healthy non-pregnant, non-lactating women, the population used in the current study, may be more stable and less likely to change compared to infants or pregnant/lactating women as the biological needs of the foetus or infant for gangliosides may be higher during these stages of life [4].

It is important to consider whether the null result may have been due to a lack of statistical power, however, this is unlikely for the following reasons. A post hoc power calculation using an average SD of 2.88 from the current study for differences between groups in plasma GM3 (data previously unknown) confirmed statistical power of 99.9% power at α = 0.05 to detect a difference of 4 mg/L (used in the prospective power calculation). The effect size of 4 mg/L is relatively large and although it may be biologically achievable as shown in pregnant women from 2^nd^ to 3^rd^ trimester [15], it is probably not achievable with a ganglioside supplement. Furthermore, the minimum effect size that may be clinically relevant under postprandial conditions is unknown. Re-calculating the statistical power using a difference of 2 mg/L (a difference closer to that observed in serum GM3 levels in the infant study described above [22]) indicated statistical power of 76%. Nevertheless, the biggest mean difference in plasma GM3 concentrations between groups in the current study was very small; 166 ng/mL (0.17 mg/L) at 0.5 h post-meal consumption. This small difference seems unlikely to be of any clinical significance. The most likely conclusion is that acute supplementation with a high GA meal does not impact GM3 concentrations in plasma. It is important to note that this conclusion cannot be extrapolated to a chronic supplementation situation where days and weeks of supplementation are undertaken.

Strengths of the current study include a rigorous randomized controlled study design. The dosage of gangliosides (112 mg) consumed in the one meal was high relative to estimated intakes from the whole diet (<200 mg/day [12]). The high dosage was well tolerated by participants with no adverse events reported after consumption of the high ganglioside meals. 

Furthermore, conclusions are limited to healthy non-pregnant, non-lactating women of child bearing age and cannot be generalized to pregnant and lactating women, men or infants. 

## 5. Conclusions, Implications and Recommendations

A growing body of scientific evidence is emerging linking gangliosides to a variety of bioactivities such as neurological development, intestinal maturation, intestinal immunity development and gut-barrier function [4,8,9,10]. While the importance of gangliosides continues to build, key knowledge gaps exist that are important for designing human studies, specifically the best approaches regarding sample type and storage of blood samples for ganglioside analysis, the normal diurnal and day-to-day variation in circulating gangliosides as well as the acute impact of consuming bovine milk derived gangliosides on circulating levels of gangliosides in humans. These gaps affect when samples should be taken during the day and whether participants must be fasted or not. This study aimed to address these key knowledge gaps. Data reported is for GM3 gangliosides, the main ganglioside in the circulation.

From the results presented, it can be concluded that either serum or plasma (specifically lithium heparin plasma) can be used for GM3 ganglioside analysis, as no differences were seen in concentrations between these two types of samples. GM3 gangliosides in plasma/serum samples stored at −20 °C and −70 °C seems to be stable for at least up to 6 months or up to 12 months for total GM3 stored at −20 °C. Before final conclusions can be made, it is recommended that future experiments include analysis at the zero-time point (within 1 week of blood collection). Although GM3 ganglioside concentrations were lower in samples stored at −70 °C compared to −20 °C, this finding requires further examination to determine whether this is truly a stability issue or an analytical method issue. Specifically, it is recommended that effects of the extraction method as well as sample defrosting time and temperature be investigated. 

Plasma samples for investigating circulating ganglioside dynamics were stored for less than 4 months at −70 °C, hence GM3 concentrations would not have been affected by storage conditions. Plasma GM3 gangliosides did not change over the course of an 8-h day or between days. Recent changes in diet (low ganglioside diet over 7 days vs. habitual diet) did not affect fasting total plasma GM3 ganglioside concentrations. Plasma GM3 gangliosides were also not affected postprandially over 8 h after consumption of a high bovine derived ganglioside meal compared to a low ganglioside meal. The implications of these findings for future intervention and epidemiological studies may be that blood samples for plasma GM3 ganglioside analysis can be taken at any time of the day as concentrations did not vary diurnally or from day-to-day. In addition, participants do not have to be in the fasted state as recent intakes of dietary gangliosides did not change circulatory concentrations. However, the findings will have to be confirmed within other target populations such as pregnant, lactating women, infants, individuals with gut disorders and men. These results cannot be extrapolated to chronic feeding and it is recommended that the effects of long-term (week/months) intake of high vs. low gangliosides be investigated. Further development and validation of the methodology to detect all ganglioside types is needed as well as standardization of ganglioside methodology across laboratories to enable high throughput analysis from clinical and epidemiological studies. The ability to analyze ganglioside precursor neutral sphingolipids such as glucosyl and lactosyl ceramides or breakdown products such as lactosylceramide may be useful in elucidating the fate of dietary gangliosides in future feeding trials.

## Figures and Tables

**Table 1:** Mean (SD) differences in GM3 gangliosides between serum and plasma samples ^1^ taken at T0 and T60 and stored at −20 and −70 °C.

GA Species (ng/mL)	°C	T0					T60				
		Plasma		Serum		*P*-Value	Plasma		Serum		*P*-Value ^2^
		Mean	SD	Mean	SD		Mean	SD	Mean	SD	
**GM3 32:1**	−20	677	129	752	180	0.17	626	142	662	146	0.52
	−70	502	117	591	126	0.06	608	146	572	209	0.65
**GM3 34:2**	−20	751	201	766	174	0.70	663	170	773	146	0.02
	−70	592	125	629	148	0.49	616	134	619	187	0.96
**GM3 34:1**	−20	4844	893	4778	948	0.92	4797	1052	4895	869	0.78
	−70	4538	1233	4426	1061	0.80	4738	1074	4614	1132	0.78
**GM3 36:2**	−20	705	200	636	164	0.44	661	139	695	181	0.54
	−70	633	138	704	225	0.32	643	193	613	226	0.69
**GM3 36:1**	−20	2056	466	1970	399	0.72	1889	521	1990	509	0.47
	−70	1848	683	2106	481	0.33	2064	500	2222	632	0.33
**GM3 38:2**	−20	405	81	332	88	<0.001	376	90	401	86	0.37
	−70	379	86	384	78	0.83	394	103	395	153	0.97
**GM3 38:1**	−20	934	249	907	242	0.66	936	276	946	218	0.86
	−70	886	158	910	181	0.70	871	235	994	274	0.21
**GM3 40:2**	−20	566	114	530	99	0.42	580	129	529	108	0.28
	−70	517	117	607	103	0.05	544	105	541	161	0.95
**GM3 40:1**	−20	987	226	875	204	0.34	1032	419	1098	246	0.55
	−70	961	176	1076	251	0.18	947	208	1201	394	0.06
**GM3 42:2**	−20	1131	396	1042	295	0.30	1123	283	1215	267	0.18
	−70	974	246	1148	351	0.18	1072	282	1157	282	0.43
**GM3 42:1**	−20	797	245	782	237	0.77	805	258	850	237	0.42
	−70	690	208	828	279	0.11	689	201	839	254	0.08
**Total GM3**	−20	13,854	2504	13,371	2239	0.69	13,489	2725	14,054	2061	0.49
	−70	12,518	2537	13,410	2373	0.42	13,186	2623	13,767	3289	0.62

(ng/mL = 0.001 mg/L). GA, gangliosides ^1^ Three months samples ^2^ Differences between serum and plasma samples analyzed using Paired Samples *T*-Test.

**Table 2:** Mean (SD) differences in plasma GM3 gangliosides between different storage times and storage temperatures ^1.^

GA Species (ng/mL)	Temp	3 months		6 months		12 months		18 months		*P*-Value ^2^		
		Mean	SD	Mean	SD	Mean	SD	Mean	SD	Time	Temp	Time * Temp
**GM3 32:1 ^3^**	−20	677	129	684	112	725 ^a^	246	800 ^b^	249	0.001	<0.001	0.31
	−70	502	117	508	120	444 ^a^	123	547 ^b^	127			
**GM3 34:2**	−20	751	201	762	127	784	180	780	216	0.57	0.001	0.19
	−70	592	125	625	96	545	118	581	96			
**GM3 34:1 ^3^**	−20	4844 ^a^	893	4837 ^a^	570	3926 ^b^	1207	3626 ^b^	913	<0.001	0.02	0.12
	−70	4538 ^a^	1233	4962 ^a^	766	3081 ^b^	443	3254 ^b^	600			
**GM3 36:2 ^3^**	−20	705 ^a^	200	597 ^a^	104	551 ^b^	128	590 ^b^	165	<0.001	0.060	0.05
	−70	633 ^a^	138	627 ^a^	116	522 ^b^	118	488 ^b^	118			
**GM3 36:1 ^3^**	−20	2056 ^a^	466	1967 ^a^	380	1536 ^b^	336	1446 ^b^	366	<0.001	0.080	0.74
	−70	1848 ^a^	683	1887 ^a^	347	1411 ^b^	202	1336 ^b^	250			
**GM3 38:2 ^3^**	−20	405 ^a^	81	297 ^b^	41	320 ^b^	77	317 ^b^	80	<0.001	0.009	0.05
	−70	379 ^a^	86	300 ^b^	47	255 ^b^	42	255 ^b^	36			
**GM3 38:1**	−20	934 ^a^	249	867 ^b^	162	839 ^b,c^	246	837 ^c^	184	<0.001	0.002	0.08
	−70	886 ^a^	158	690 ^b^	113	612 ^b,c^	152	556 ^c^	157			
**GM3 40:2**	−20	566 ^a^	114	801 ^b,^*	255	857 ^b,^*	130	886 ^b,^*	201	0.001	0.001	0.002
	−70	517	117	548 ^#^	93	632 ^#^	61	620 ^#^	59			
**GM3 40:1 ^3^**	−20	987 ^a^	226	876 ^a,^*	137	616 ^b,^*	130	698 ^b,^*	168	<0.001	<0.001	<0.001
	−70	961 ^a^	176	647 ^b,#^	99	390 ^c,#^	72	422 ^c,#^	55			
**GM3 42:2 ^3^**	−20	1131	396	930 ^a,*^	240	1401 ^*^	430	1745 ^b,*^	564	<0.001	<0.001	0.001
	−70	974 ^a^	246	679 ^b,#^	156	876 ^a,#^	179	983 ^#^	248			
**GM3 42:1 ^3^**	−20	797 ^a^	245	469 ^b,*^	114	485 ^b,*^	141	605 ^b,*^	223	<0.001	<0.001	<0.001
	−70	690 ^a^	208	362 ^b,#^	93	306 ^c,#^	116	307 ^c,#^	97			
**Total GM3 ^3^**	−20	13,854	2504	13,087 *	1664	12,041 *	2669	12,329 *	2475	<0.001	0.001	0.001
	−70	12,518 ^a^	2537	11,836 ^a,#^	1466	9075 ^b,#^	945	9349 ^b,#^	1244			

(ng/mL = 0.001 mg/L). GA, gangliosides ^1^ Plasma Samples taken at T0. ^2^ Comparisons over storage periods and storage temperatures and interactions between storage times and temperatures were analyzed using mixed effects longitudinal models. ^3^ Variables log transformed to achieve greater model validity ^a,b, *, #^ In cases where significant (*P* < 0.05) time*temperature interactions were shown, post-hoc analyses were conducted stratified for storage time and temperature and Bonferroni adjustments made. Different symbols differed significantly, ^a,b^—post-hoc analysis over storage times; ^*,#^—post-hoc analyses over storage temperatures.

**Table 3:** Diurnal variability in plasma GM3 gangliosides (*n* = 61).

Ganglioside Species (ng/mL)	Hours												*P*-Value (Time) ^1^
	0		1		2		4		6		8		
	Mean	SD	Mean	SD	Mean	SD	Mean	SD	Mean	SD	Mean	SD	
**GM3 32:1**	266	96.5	260	97.3	253	89.0	270	94.0	264	96.9	253	83.6	0.18
**GM3 34:2**	346	109	355	106	341	111	348	112	351	115	333	108	0.41
**GM3 34:1**	2601	644	2590	670	2515	621	2549	575	2577	658	2549	645	0.74
**GM3 36:2**	376	109	378	114	353	110	365	118	371	105	363	117	0.08
**GM3 36:1**	1135	288	1147	294	1116	300	1130	342	1102	323	1086	280	0.12
**GM3 38:2**	206	63.5	205	64.5	202	71.2	209	59	206	65.1	206	69.6	0.82
**GM3 38:1**	385	132	413	156	397	160	400	154	402	156	396	154	0.27
**GM3 40:2**	223	63.8	225	82.7	223	80.2	225	66.6	232	82.0	219	77.1	0.50
**GM3 40:1**	390 ^a^	105	441 ^b^	143	421	142	411	123	423	117	420	124	0.04
**GM3 42:2**	398 ^a^	108	430 ^b^	127	426	132	427	119	421	130	422	125	0.04
**GM3 42:1**	292 ^a^	98.7	309	97.2	301	106	313	112	315 ^b^	98.7	312	92.2	0.02
**Total GM3**	6619	1453	6751	1606	6548	1594	6648	1532	6663	1584	6560	1509	0.74

(ng/mL = 0.001 mg/L) GA, gangliosides ^1^ Comparisons between time-points were made on log transformed data using mixed effects models ^a,b^—Values with different superscript letters indicate significant differences (Post-hoc analysis with Bonferroni adjustments

**Table 4:** Day-to-day variability in plasma GM3 gangliosides (*n* = 30).

GA Species (ng/mL)		Hours						*P*-Value ^1^		
		0		1		2		Time	Day	Time * Day
	Day	Mean	SD	Mean	SD	Mean	SD			
**GM3 32:1**	Day 1	286	95.4	298	114	320 ^a^	116	0.62	0.42	0.05
	Day 2	303	142	302	117	281 ^b^	98.2			
**GM3 34:2**	Day 1	339	61.8	340	101	384	126	0.41	0.32	0.09
	Day 2	344	97.2	353	98.9	334	73.6			
**GM3 34:1**	Day 1	2571	502	2591	509	2812 ^a^	620	0.68	0.06	0.02
	Day 2	2559	578	2624	721	2427 ^b^	510			
**GM3 36:2**	Day 1	344	98.9	312 ^b^	76.4	358 ^a^	111	0.56	0.83	0.01
	Day 2	339	94.8	349	113	318 ^b^	73.6			
**GM3 36:1**	Day 1	1071	266	1031	254	1110	305	0.84	0.15	0.08
	Day 2	1021	231	1042	247	998	220			
**GM3 38:2**	Day 1	184	51.9	174	51.2	195	63.0	0.63	0.46	0.25
	Day 2	181	43.9	180	62.4	174	49.2			
**GM3 38:1**	Day 1	408	90.2	411	99.5	442 ^a^	126	0.76	0.07	0.04
	Day 2	388	85.8	404	110	375 ^b^	104			
**GM3 40:2**	Day 1	243	73.9	235	58.5	256	58.7	0.10	0.11	0.8
	Day 2	226	57.2	230	68.5	236	45.2			
**GM3 40:1**	Day 1	425 ^a^	152	438 ^b^	162	458	228	0.01	0.24	0.33
	Day 2	386 ^a^	108	447 ^b^	148	409	137			
**GM3 42:2**	Day 1	438	140	450	167	470	173	0.04	0.46	0.39
	Day 2	411	122	452	139	430	96.6			
**GM3 42:1**	Day 1	270	112	282	125	300	174	0.23	0.57	0.87
	Day 2	262	106	277	103	273	92.4			
**Total GM3**	Day 1	6578 ^b^	1110	6562	1192	7106^a^	1665	0.40	0.06	0.03
	Day 2	6421	1234	6661	1531	6255^b^	963			

(ng/mL = 0.001 mg/L). GA, gangliosides ^1^ Comparisons between time-points were made on log transformed data using mixed effects models. ^a,b^—Values with different superscript letters indicate significant differences between days and over time (Post-hoc analysis with Bonferroni adjustments.

**Table 5:** Differences in fasting plasma GM3 gangliosides after consuming a habitual diet vs. a low ganglioside diet for a week.

GA Species (ng/mL)	Habitual Diet (*n* = 61)		Low GA Diet (*n* = 61)		*P*-Value ^1^
	Mean	SD	Mean	SD	
**GM3 32:1**	266	96.5	244	79.3	0.03
**GM3 34:2**	346	109	341	111	0.64
**GM3 34:1**	2601	644	2570	615	0.63
**GM3 36:2**	376	109	345	103	0.006
**GM3 36:1**	1135	288	1109	305	0.28
**GM3 38:2**	206	63.5	215	65.8	0.15
**GM3 38:1**	385	132	403	158	0.09
**GM3 40:2**	223	63.8	249	70.6	0.001
**GM3 40:1**	390	105	445	146	0.001
**GM3 42:2**	398	108	426	107	0.01
**GM3 42:1**	292	98.7	339	106	<0.001
**Total GM3**	6619	1453	6685	1484	0.62

(ng/mL = 0.001 mg/L) GA, gangliosides ^1^ Differences between groups analyzed using Paired Samples *T*-Test.

**Table 6:** Changes in plasma GM3 gangliosides over 8 h after consumption of either a high- or a low ganglioside meal.

Meal	GA Species (ng/mL)	Hours														*P*-Value ^1^		
		0		0.5		1		2		4		6		8				
		Mean	SD	Mean	SD	Mean	SD	Mean	SD	Mean	SD	Mean	SD	Mean	SD	Meal	Time	Time * Meal
**A**	GM3 32:1	249	91.4	236	71.0	233	97.0	231	75.7	241	80.7	232	80.8	229	71.2	0.86	0.62	0.75
**B**		239	67.7	236	70.8	232	76.3	235	74.5	228	69.6	226	74.4	238	65.1			
**A**	GM3 34:2	345	128	327	113	334	118	326	102	330	117	327	100	337	121	0.85	0.48	0.9
**B**		338	96.1	321	104	314	96.1	333	105	321	98	314	94.9	330	93.3			
**A**	GM3 34:1	2631 ^a^	651	2562	570	2437 ^b^	681	2397	564	2401 ^b^	611	2389 ^b^	495	2447	621	0.66	0.02	0.48
**B**		2514 ^a^	585	2388	576	2346 ^b^	499	2401	485	2361 ^b^	490	2337 ^b^	545	2422	540			
**A**	GM3 36:2	358	106	353	103	356 ^a^	103	331	114	325 ^b^	103	319	95.0	338	103	0.69	0.004	0.18
**B**		333	100	334	89.8	338 ^a^	89.5	333	89.2	321 ^b^	85.1	322	85.5	314	93.9			
**A**	GM3 36:1	1132 ^a^	357	1113	298	1078	319	1075	298	1062 ^b^	310	1065	277	1074	285	0.58	0.002	0.79
**B**		1087 ^a^	254	1065	262	1039	270	1062	279	990 ^b^	250	1009	309	1032	293			
**A**	GM3 38:2	218 ^a^	64.3	214 ^a^	71.6	213	67.1	196	63.3	201 ^b^	61.7	212	63.9	202	74.3	0.75	<0.001	0.24
**B**		213 ^a^	68.0	214 ^a^	62.9	206	63.2	204	60.5	187 ^b^	57.2	196	59.8	194	60.4			
**A**	GM3 38:1	385	129	399	127	397	115	410	123	402	113	396	115	407	141	0.57	0.18	0.73
**B**		418	182	411	165	428	196	453	187	410	161	435	190	428	194			
**A**	GM3 40:2	240 ^a^	67.4	221 ^b^	85.4	217	60.2	217 ^b^	69.7	229	85.4	215 ^b^	65.4	217 ^b^	58.3	0.31	0.004	0.95
**B**		256 ^a^	73.7	229 ^b^	73.4	242	75.8	234 ^b^	80.0	231	58.7	231 ^b^	69.4	241 ^b^	88.2			
**A**	GM3 40:1	444	148	426	144	427	149	421	145	444	159	446	171	464	171	0.37	0.1	0.51
**B**		446	146	437	123	448	142	464	132	453	104	486	130	487	165			
**A**	GM3 42:2	426	103	409	143	421	125	413	125	427	149	445	164	446	139	0.40	0.17	0.52
**B**		427	112	456	150	432	128	444	132	456	121	477	143	457	143			
**A**	GM3 42:1	338	109	317	116	320	121	330	123	320	119	337	144	339	135	0.71	0.12	0.95
**B**		341	106	320	104	329	111	324	94	332	116	352	124	336	106			
**A**	Total GM3	6765	1604	6577	1538	6433	1628	6348	1457	6384	1582	6382	1452	6502	1593	0.99	0.16	0.84
**B**		6613	1388	6411	1297	6353	1354	6488	1295	6290	1154	6385	1379	6478	1439			

(ng/mL = 0.001 mg/L) GA, gangliosides. Group A = high ganglioside meal, n=29; Group B = low ganglioside meal, *n* = 32. ^1^ Comparisons between meals over time were made on log transformed data using mixed effects models. Values with different superscript letters indicate significant differences (Post-hoc analysis with Bonferroni adjustments.

## Supplementary Materials

The following are available online at https://www.mdpi.com/2072-6643/12/3/711/s1, Figure S1: Chromatogram illustrating a participant’s MS results, Figure S2: Individual participant differences in total GM3 (mg/mL) between serum and plasma samples (samples at 3 month, T0, −20 °C), Figure S3: Individual participant changes in plasma total GM3 gangliosides stored over time at −20 °C (A) and −70 °C (B), Figure S4: Changes in individual participant’s total plasma GM3 concentrations over 8 h (visit 2), Figure S5: Changes in individual participant’s total plasma GM3 concentrations over 8 h after consumption of either high- (A, *n* = 29) or low (B, *n* = 32) ganglioside meals (visit 3); Table S1: MS conditions for GM3 ganglioside analysis.

## References

[1] Zheng L., Fleith M., Giuffrida F., O’Neill B.V., Schneider N. (2019). Dietary Polar Lipids and Cognitive Development: A Narrative Review. Adv. Nutr..

[2] Svennerholm L. (1963). Chromatographic separation of human brain gangliosides. J. Neurochem..

[3] Fong B.Y., Ma L., Khor G.L., van der Does Y., Rowan A., McJarrow P. (2016). Ganglioside Composition in Beef, Chicken, Pork, and Fish Determined Using Liquid Chromatography-High-Resolution Mass Spectrometry. J. Agric. Food Chem..

[4] McJarrow P., Schnell N., Jumpsen J., Clandinin T. (2009). Influence of dietary gangliosides on neonatal brain development. Nutr. Rev..

[5] Schnaar R.L. (2019). The Biology of Gangliosides. Adv. Carbohydr. Chem. Biochem..

[6] Park E.J., Suh M., Ramanujam K., Steiner K., Begg D., Clandinin M.T. (2005). Diet-induced changes in membrane gangliosides in rat intestinal mucosa, plasma and brain. J. Pediatr. Gastroenterol. Nutr..

[7] Schnaar R.L. (2016). Gangliosides of the Vertebrate Nervous System. J. Mol. Biol..

[8] Rueda R. (2007). The role of dietary gangliosides on immunity and the prevention of infection. Br. J. Nutr..

[9] Yu R.K., Tsai Y.T., Ariga T. (2012). Functional roles of gangliosides in neurodevelopment: An overview of recent advances. Neurochem. Res..

[10] Miklavcic J.J., Shoemaker G.K., Schnabl K.L., Larsen B.M.K., Thomson A.B.R., Mazurak V.C. (2017). Ganglioside Intake Increases Plasma Ganglioside Content in Human Participants. JPEN J. Parenter. Enter. Nutr..

[11] Subar A.F., Kirkpatrick S.I., Mittl B., Zimmerman T.P., Thompson F.E., Bingley C. (2012). The Automated Self-Administered 24-h dietary recall (ASA24): A resource for researchers, clinicians, and educators from the National Cancer Institute. J. Acad. Nutr. Diet..

[12] Pham P.H., Duffy T.L., Dmytrash A.L., Lien V.W., Thomson A.B., Clandinin M.T. (2011). Estimate of dietary ganglioside intake in a group of healthy Edmontonians based on selected foods. J. Food Compos. Anal..

[13] Rivas-Serna I.M., Polakowski R., Shoemaker G.K., Mazurak V.C., Clandinin M.T. (2015). Profiling gangliosides from milk products and other biological membranes using LC/MS. J. Food Compos. Anal..

[14] Senn H.J., Orth M., Fitzke E., Wieland H., Gerok W. (1989). Gangliosides in normal human serum. Concentration, pattern and transport by lipoproteins. Eur. J. Biochem..

[15] Ma L., MacGibbon A.K.H., Mohamed H., Loy S., Rowan A., McJarrow P. (2015). Determination of ganglioside concentrations in breast milk and serum from Malaysian mothers using a high performance liquid chromatography-mass spectrometry-multiple reaction monitoring method. Int. Dairy J..

[16] Dunn W.B., Broadhurst D., Begley P., Zelena E., Francis-McIntyre S., Anderson N. (2011). Procedures for large-scale metabolic profiling of serum and plasma using gas chromatography and liquid chromatography coupled to mass spectrometry. Nat. Protoc..

[17] Evans K., Mitcheson J., Laker M.F. (1997). Effect of storage at −70 degrees C on lipid, lipoprotein and apolipoprotein concentrations. Clin. Chim. Acta.

[18] Hodson L., Skeaff C.M., Wallace A.J., Arribas G.L. (2002). Stability of plasma and erythrocyte fatty acid composition during cold storage. Clin. Chim. Acta.

[19] Metherel A.H., Stark K.D. (2016). The stability of blood fatty acids during storage and potential mechanisms of degradation: A review. Prostaglandins Leukot. Essent. Fat. Acids.

[20] Schauer R., Kelm S., Reuter G., Roggentin P., Shaw L., Rosenberg A. (1995). Chapter 2: Biochemistry and Role of Sialic acids. Biology of the Sialic Acids.

[21] Huang Q., Zhou X., Liu D., Xin B., Cechner K., Wang H. (2014). A new liquid chromatography/tandem mass spectrometry method for quantification of gangliosides in human plasma. Anal. Biochem..

[22] Gurnida D.A., Rowan A.M., Idjradinata P., Muchtadi D., Sekarwana N. (2012). Association of complex lipids containing gangliosides with cognitive development of 6-month-old infants. Early Hum. Dev..

[23] Meikle P.J., Barlow C.K., Mellett N.A., Mundra P.A., Bonham M.P., Larsen A. (2015). Postprandial Plasma Phospholipids in Men Are Influenced by the Source of Dietary Fat. J. Nutr..

