# Supplementary material for: Exploring In Vivo Dynamics of Bovine Milk Derived Gangliosides
Source: Nutrients. 2020 Mar 7;12(3):711. doi: 10.3390/nu12030711 (PMC7146146; doi:10.3390/nu12030711)
Supplement: Supplementary file 1 [file nutrients-12-00711-s001.pdf]

nutrients

MDPI

*Article*

# Exploring in vivo Dynamics of Bovine Milk Derived Gangliosides

**Welma Stonehouse[1,*], Bradley Klingner[1], Paul McJarrow[2], Bertram Fong[2] and Nathan O'Callaghan[1]**

## Supplementary Materials

**Table S1. MS conditions for GM3 ganglioside analysis**

| Ganglioside Species | MRM | Retention Time (min) |
|---|---|---|
| $GM_3$ (32:1) | 1123.7→290.1 | 2.02 |
| $GM_3$ (34:2) | 1149.7→290.1 | 2.13 |
| $GM_3$ (34:1) | 1151.7→290.1 | 2.57 |
| $GM_3$ (36:2) | 1177.7→290.1 | 2.70 |
| $GM_3$ (36:1) | 1179.7→290.1 | 3.20 |
| $GM_3$ (38:2) | 1205.8→290.1 | 3.32 |
| $GM_3$ (38:1) | 1207.7→290.1 | 3.85 |
| $GM_3$ (40:2) | 1233.8→290.1 | 4.00 |
| $GM_3$ (40:1) | 1235.8→290.1 | 4.70 |
| $GM_3$ (42:2) | 1261.8→290.1 | 4.80 |
| $GM_3$ (42:1) | 1263.8→290.1 | 5.30 |

MRM, m**ultiple reaction** m**onitoring**

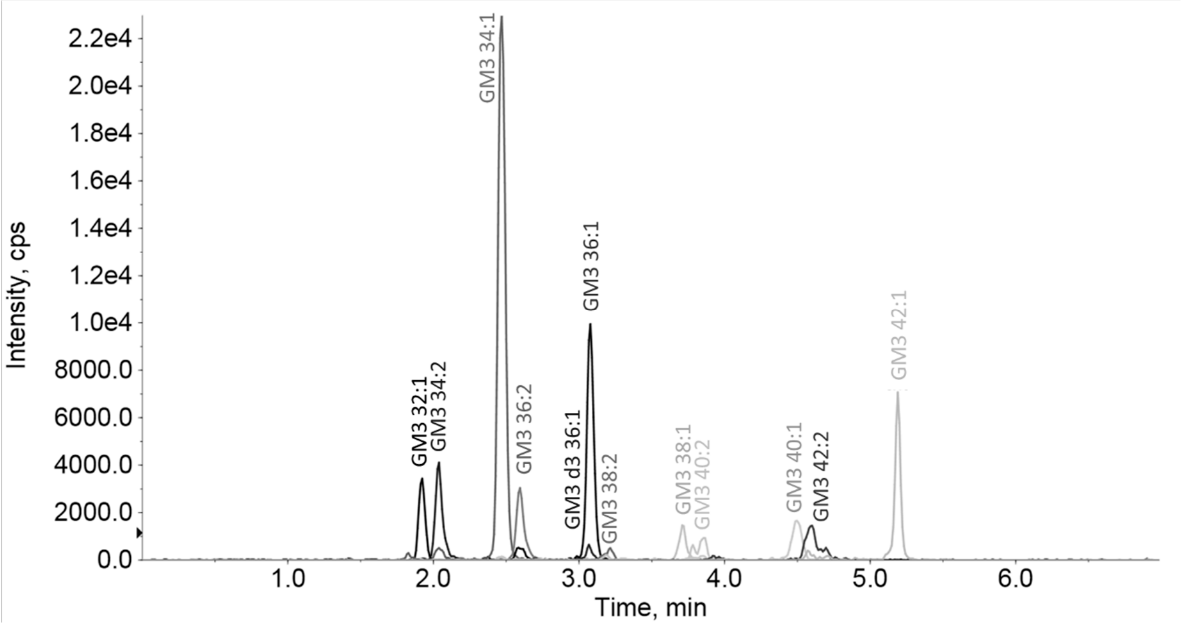


**Figure S1. Chromatogram illustrating a participant's MS results**

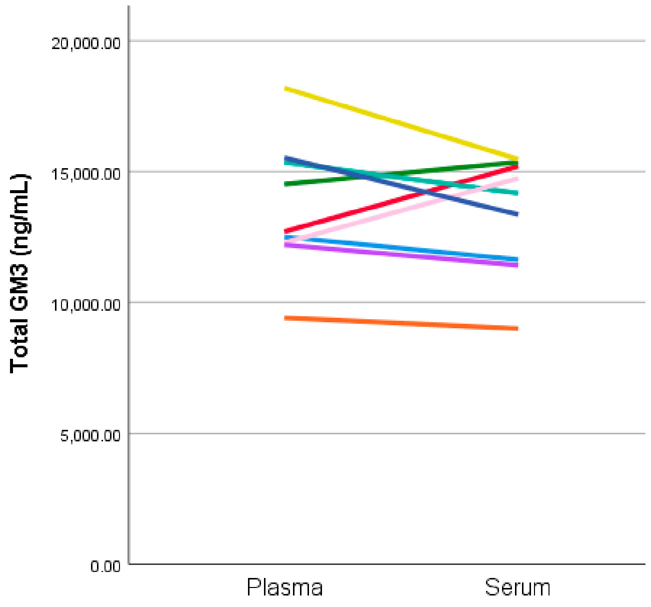


**Figure S2. Individual participant differences in total GM3 (mg/mL) between serum and plasma samples (samples at 3 months, T0, -20°C)**

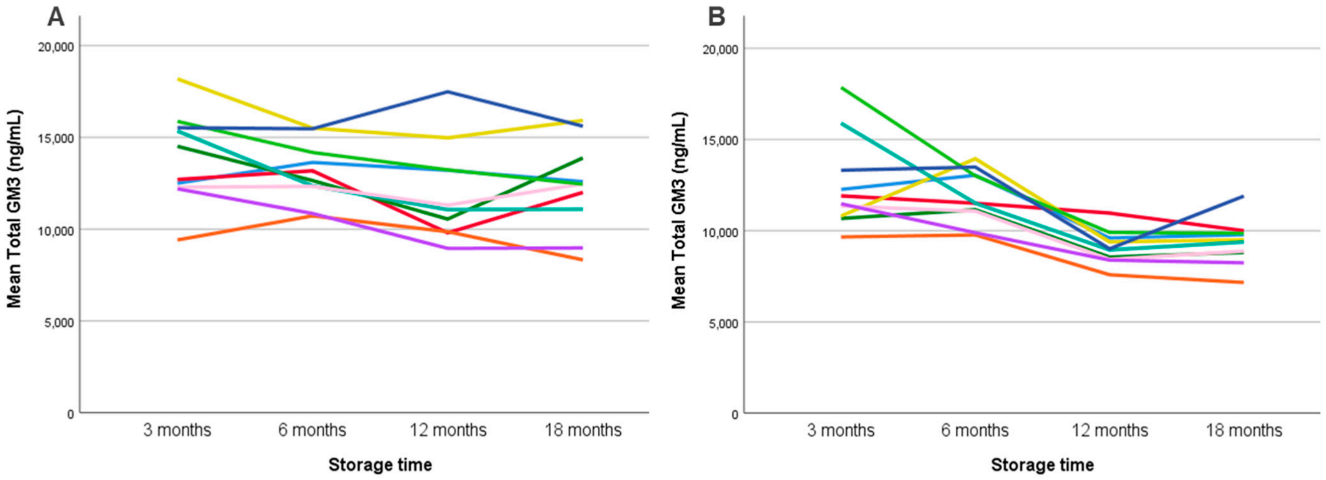


**Figure S3. Individual participant changes in plasma total GM3 gangliosides stored over time at -20◦C (A) and -70◦C (B)**

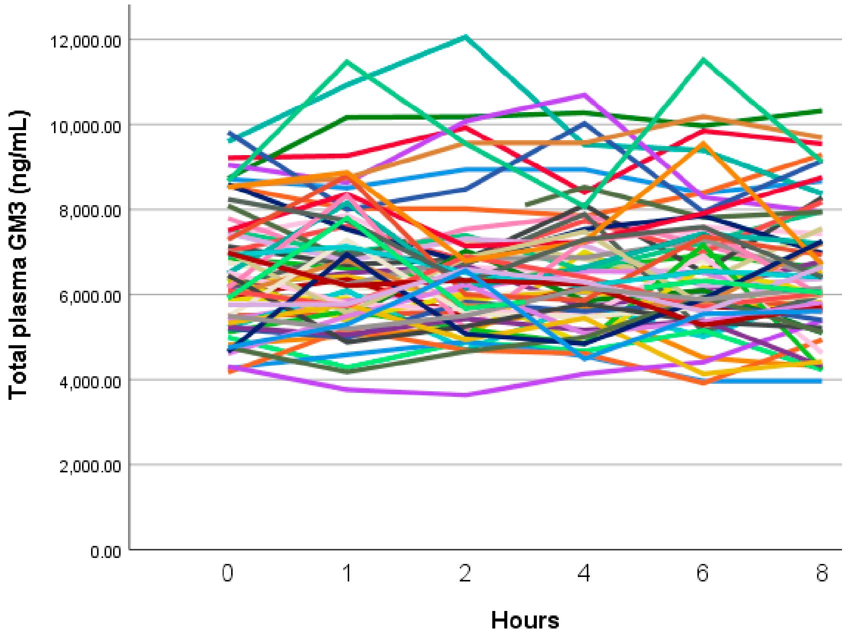


**Figure S4. Changes in individual participant's total plasma GM3 concentrations over 8 hours (visit 2)**

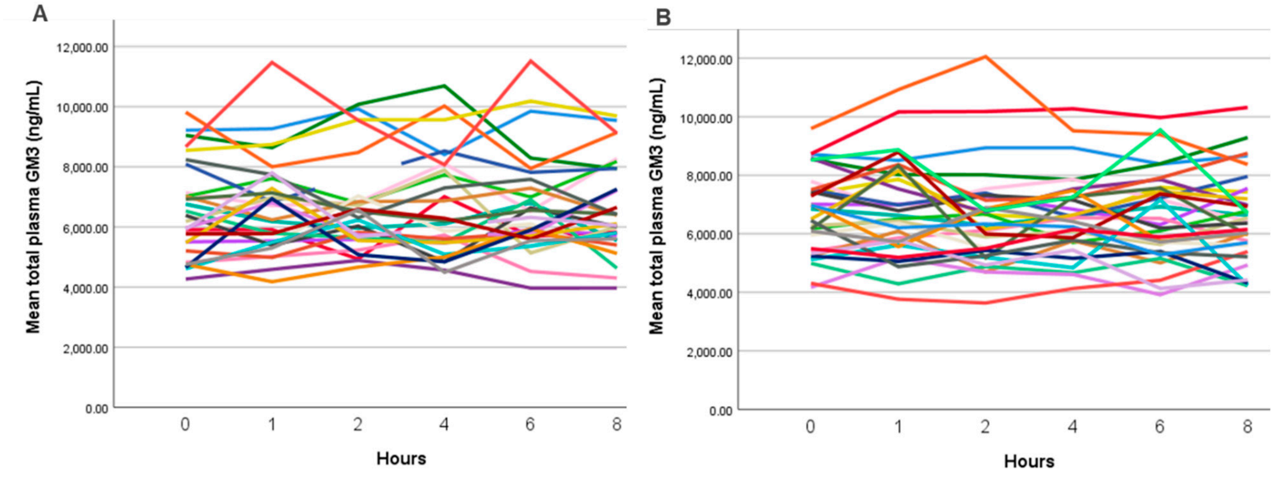


**Figure S5. Changes in individual participant's total plasma GM3 concentrations over 8 hours after consumption of either high- (A, n=29) or low (B, n=32) ganglioside meals (visit 3)**
